# *Lysimachia fanii*, a new species of Primulaceae from limestone area of Guangxi, China

**DOI:** 10.3897/phytokeys.130.34655

**Published:** 2019-08-29

**Authors:** Yun-Feng Huang, Li-Na Dong, Wei-Bin Xu

**Affiliations:** 1 Guangxi Key Laboratory of Traditional Chinese Medicine Quality Standards, Guangxi Institute of Chinese Medicine & Pharmaceutical Sciences, Nanning 530022, Guangxi, China Guangxi Institute of Chinese Medicine & Pharmaceutical Sciences Nanning China; 2 Guangxi Key Laboratory of Plant Conservation and Restoration Ecology in Karst Terrain, Guangxi Institute of Botany, Guangxi Zhuang Autonomous Region and Chinese Academy of Sciences, Guilin 541006, Guangxi, China Guangxi Institute of Botany, Guangxi Zhuang Autonomous Region and Chinese Academy of Sciences Guilin China

**Keywords:** *
Lysimachia
*, subgen. *Idiophyton*, Primulaceae, taxonomy, limestone flora

## Abstract

*Lysimachia fanii*, a new species of Lysimachia (Subgen. Idiophyton, Primulaceae), is described and illustrated from Guangxi, China based on morphological and molecular data. *Lysimachia fanii* differs from *L. verbascifolia*, *L. rupestris* and *L. alpestris* mainly by the habit being nearly rosulate, leaves congested at the apex of the rhizome, leaf blades spatulate to narrowly oblanceolate and flowers solitary. Phylogenetic analyses supported *L. verbascifolia* as sister to *L. fanii*. This new species is endemic to limestone areas in Liucheng county of Guangxi, China.

## Introduction

The genus *Lysimachia* L. (1753: 146) includes about 190 species and was originally placed in Primulaceae (Cronquist 1981, Takhtajan 1997), but later transferred into Myrsinaceae, based on morphological and molecular evidence (Anderberg and Ståhl 1995, Anderberg et al. 1998, 2002, Källersjö et al. 2000, Hao et al. 2004). Mysinaceae was later merged into Primulaceae s.l., hence *Lysimachia* was replaced into Primulaceae (China Phylogeny Consortium 2016). The majority of species within the genus are distributed in temperate and subtropical regions of the Northern Hemisphere, with some species in Africa, Australia and South America. In China, the genus has 138 species (Hu and Kelso 1996) and is highly diversified in south-western China, especially in limestone areas. According to the flower and gland morphology, the genus is separated into five subgenera, viz. subgen. Idiophyton Hand.-Mazz., subgen. Lysimachia, subgen. Palladia (Moench) Hand.-Mazz., subgen. Heterostylandra (Hand.-Mazz.) F.H.Chen & C.M.Hu and subgen. Naumburgia (Moench) Klatt. (Chen and Hu 1979, Chen et al. 1989).

The south-western limestone karst area is one of China’s biodiversity hotspots. These areas are fragile and sensitive to environmental change and, in the wake of the rapid economic development of China, they are facing serious threat. Documentation of the plant diversity in these regions is urgently needed. Thus, we are surveying traditional medicinal plants in the limestone areas of Guangxi and trying to increase our knowledge of these poorly studied areas. During fieldwork in May 2018, we discovered an unknown species in *Lysimachia*. This species is allied to subgen. Heterostylandra by having rosette leaves, but it differs in having heteromorphic flowers. It shows alliance to subgen. Idiophyton, subgen. Lysimachia and subgen. Palladia by having 5-merous flowers, but has unique filaments, anthers and glands. After morphological observation and consulting relevant literature (Chen and Hu 1979, Chen et al. 1989, Hu and Kelso 1996, Tong et al. 2017), we confirm that the rare plant is a new species and has been placed into subgen. Idiophyton, based on morphology and molecular analyses.

## Material and methods

### Taxon sampling

We followed the classification of *Lysimachia* of Chen et al. (1989) and Hu and Kelso (1996). Leaves were collected from the holotype (L.Y. Fan et al., FLY2018001 in GXMI) and paratypes (L.Y. Fan et al., FLY2018002 in IBK & GXMI) to represent the new species. Twenty related taxa within subgen. Idiophyton, one taxon within subgen. Heterostylandra and four taxa within subgen. Lysimachia were selected to ascertain the phylogenetic relationships within *Lysimachia* (Anderberg et al. 2002). Based on Yan et al. (2018), *Pelletiera verna* A. St.-Hil. and *Anagallis monelli* L. were selected as outgroups.

### DNA sequencing

Total genomic DNA was extracted from silica-dried plant leaves by a modified CTAB protocol (Doyle and Doyle 1987). Four chloroplast DNA regions (*atp*F-*atp*H, *rpl*32-*trn*L, *trn*L-F and *trn*S-*trn*G) and one nuclear loci (ITS) were selected and amplified following Yan et al. (2018). Genebank Accession Numbers are listed in Table 1.

**Table 1.** Species of *Lysimachia* and related taxa sampled and GenBank accession numbers of sequences used in this study.

Taxa	*atp*F-*atp*H	*rpl*32-*trn*L	*trn*L-F	*trn*S-*trn*G	ITS
*Anagallis monelli*	MG950735	MG950945	MG951268	MG951373	MG877752
*L. alpestris*	MG950743	MG950953	MG951276	MG951381	MG877760
*L. baviensis*	MG950773	MG950983	MG951306	MG951410	MG877790
*L. capillipes*	MG950748	MG950958	MG951281	MG951386	MG877765
*L. chapaensis*	MG950749	MG950959	MG951282	MG951387	MG877766
*L. confertifolia*	MG950757	MG950967	MG951290	–	MG877774
*L. crispidens*	MG950759	MG950969	MG951292	MG951396	MG877776
*L. engleri*	MG950765	MG950975	MG951298	MG951402	MG877782
*L. foenum-graecum*	MG950770	MG950980	MG951303	MG951407	MG877787
*L. heterobotrys*	MG950779	MG950989	MG951311	MG951415	MG877796
*L. insignis*	MG950784	MG950994	MG951316	MG951420	MG877801
*L. lancifolia*	MG950788	MG960998	MG951320	MG951424	MG877805
*L. laxa*	MG950789	MG950999	MG951321	MG951425	MG877806
*L. longipes*	MG950792	MG951002	MG951324	MG951428	MG877809
*L. microcarpa*	MG950796	MG951006	MG951328	MG951432	MG877813
*L. millietii*	MG950797	MG951007	MG951329	MG951433	MG877814
*L. nemorum*	MG950799	MG951009	MG951331	MG951435	MG877816
*L. nutantiflora*	MG950801	MG951011	MG951333	MG951437	MG877818
*L. peduncularis*	MG950805	MG951015	MG951337	–	MG877822
*L. petelotii*	MG950808	MG951018	MG951340	–	MG877825
*L. pittosporoides*	MG950810	MG951020	MG951342	MG951445	MG877827
*L. punctata*	MG950813	MG951023	MG951345	MG951448	MG877830
*L. trichopoda*	MG950826	MG951038	MG951359	MG951461	MG877845
*L. verbascifolia*	MG950827	MG951039	MG951360	MG951462	MG877846
*L. vittiformis*	MG950828	MG951040	MG951361	MG951463	MG877847
*L. vulgaris*	MG950829	MG951041	MG951362	MG951464	MG877848
*Pelletiera verna*	MG950832	MG951044	MG951365	MG951467	MG877851
*L. fanii* 01	MK516268	MK516270	MK516272	–	MK516275
*L. fanii* 02	MK516269	MK516271	MK516273	MK516274	MK516276

### Phylogenetic analysis

Sequences of each DNA region were aligned using MUSCLE 3.8.31 (Edgar 2004a, 2004b) and adjusted manually where necessary. Indels were treated as gaps and all regions were combined as a single region for further study.

Maximum Parsimony (MP) analyses were conducted using PAUP v.4.0b10 (Swofford 2002). Heuristic searches were carried out with 1000 replicates and tree-bisection-reconnection (TBR) branch swapping. A strict consensus tree was summarised from all the most parsimonious trees. Node support was assessed by 500 bootstrap replicates using TBR branch swapping.

Bayesian Inference (BI) analyses were conducted using MrBayes version 3.1.2 (Ronquist and Huelsenbeck 2003). The Markov chain Monte Carlo (MCMC) chains were run for 100 000 generations while trees were sampled every 100 generations. The MCMC chains were stopped when the average standard deviation of the split frequencies was 0.008 after 100 000 generations, which meant that the chains were converged to a stationary distribution. A majority-rule consensus tree was constructed after removing a burn-in of 25% of the trees. Posterior Probability (PP) values were used to estimate branch support.

## Results

### Molecular systematic relationship

**Figure 1.**
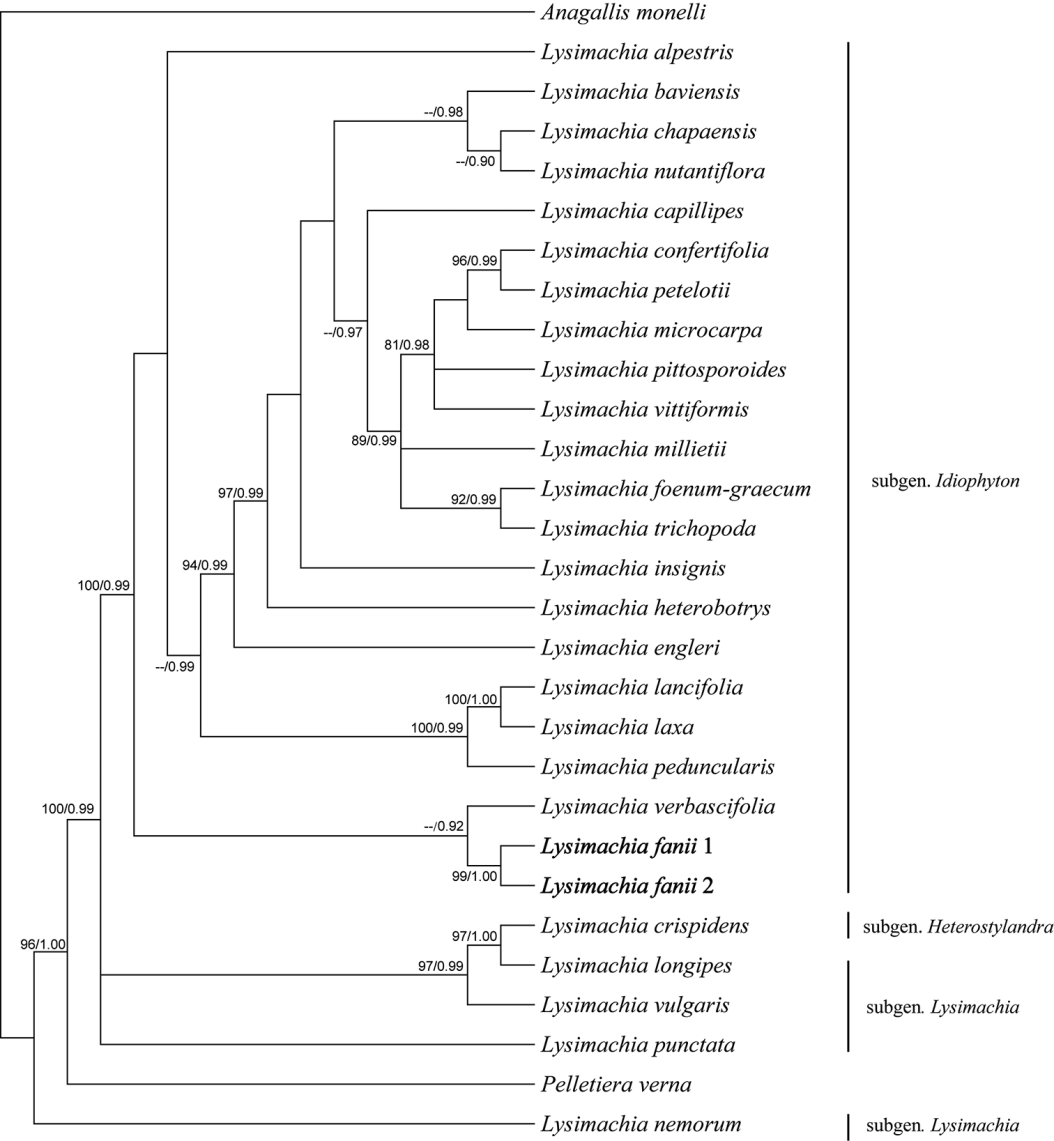
Phylogenetic tree inferred by MP and BI analyses based on the combined dataset of four plastid loci (*atp*F-*atp*H, *rpl*32-*trn*L, *trn*L-F and *trn*S-*trn*G) and nuclear ITS. Numbers above branches indicate maximum parsimony bootstrap/Bayesian inference posterior probability.

In total, 29 *atp*F-*atp*H, *rpl*32-*trn*L, *trn*L-*trn*F and ITS sequences and 25 *trn*S-G sequences were included. The combined matrix has a length of 3649 aligned characters (ITS: 653bp, *atp*F-*atp*H: 512bp, *rpl*32-*trn*L: 728bp, *trn*L-*trn*F: 946bp, *trn*S-G: 810bp), of which 363 are parsimony informative. The inferred phylogenies using MP and BI analyses are congruent (Fig. 1). The two samples of the new species (*L. fanii*) are clustered into subgenus Idiophyton with strong support values in both MP and BI analyses (BS= 100%, PP = 0.99). *L. verbascifolia* is placed as the sister group to *L. fanii* with high support in the BI analysis (PP = 0.92).

### Taxonomic treatment

#### Lysimachia fanii

Taxon classificationPlantaeEricalesPrimulaceae

Y.Feng Huang, W.B.Xu & L.N.Dong sp. nov.

AB9F876A4ABB544C960517FADA444ECD

urn:lsid:ipni.org:names:60479343-2

Figs 2 , 3

##### Type.

CHINA. Guangxi Zhuangzu Autonomous Region: Liucheng County, Taiping Town, 23°42'50"N, 109°29'20"E, 320 m a.s.l., 21 May 2018, flowering, *L.Y. Fan et al. FLY2018001* (holotype, GXMI!; isotypes, IBK!, GXMI!).

##### Diagnosis.

*Lysimachia fanii* differs from congeneric species in subgen. Idiophyton mainly by the habit being nearly rosulate, leaves congested at the apex of the rhizome, leaf blades spatulate to narrowly oblanceolate and flowers being solitary.

**Figure 2.**
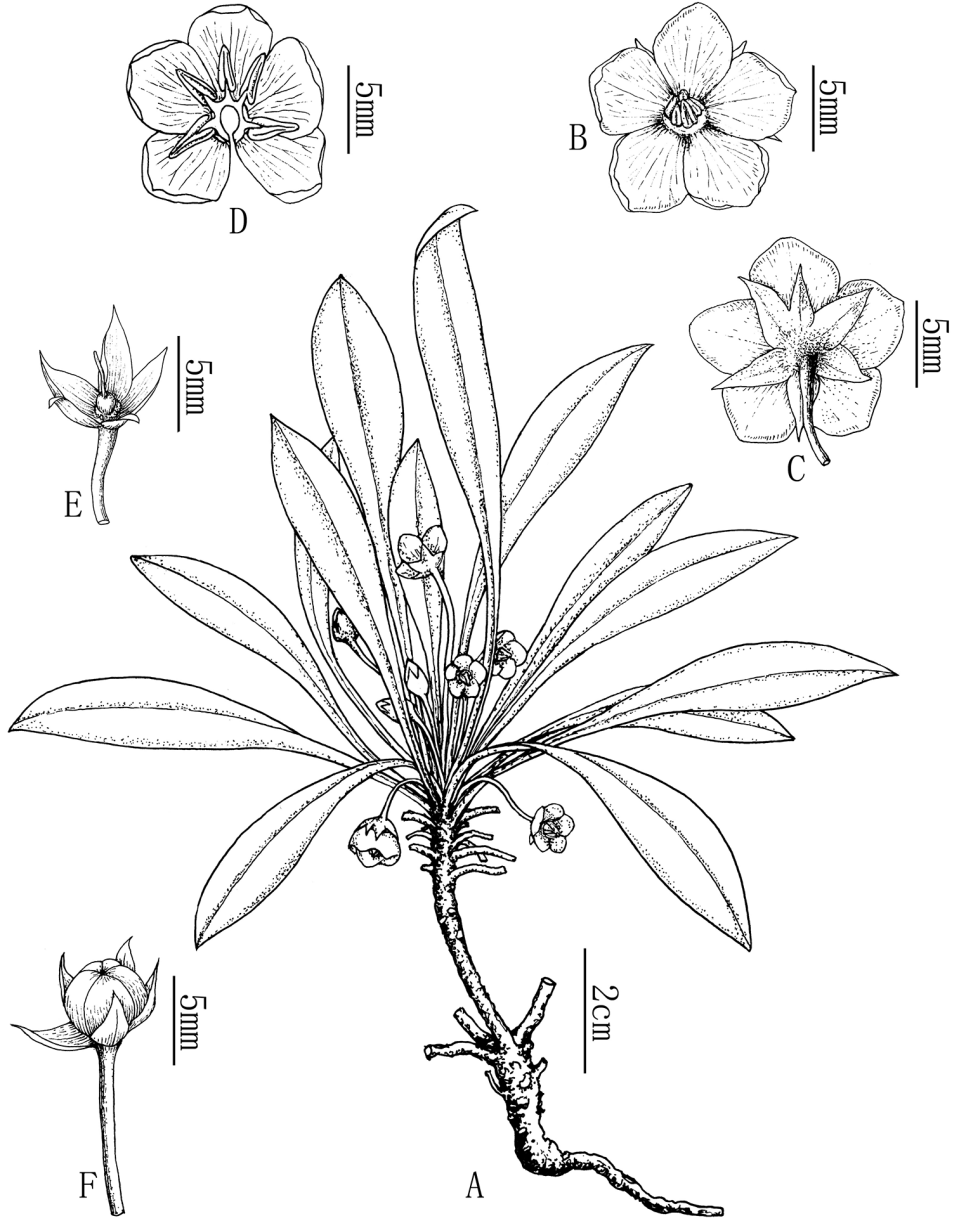
*Lysimachia fanii*. **A** Habit **B** flower, frontal view **C** flower, back view (showing six calyx lobes) **D** corolla opened showing stamens **E** calyx and pistil **F** capsule. (Drawn by X.C. Qu from the holotype).

**Figure 3.**
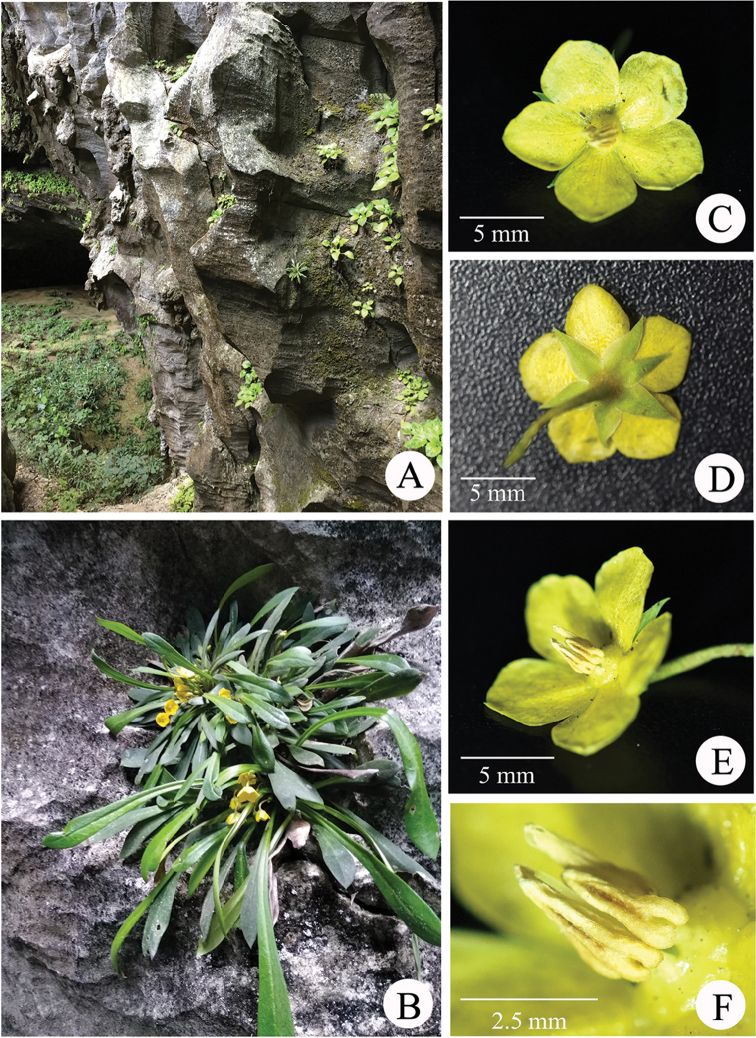
*Lysimachia fanii*. **A** Habitat **B** habit **C** flower, frontal view **D** flower, back view **E** flower, lateral view **F** stamens.

##### Description.

Herbaceous perennial, glabrous. Rhizome subterete, 6–8 cm long, 4–6 mm in diameter, branched at the apex of the rhizome. Leaves papery, thickly papery to thinly leathery when dry, spirally arranged, congested at the apex of the rhizome, ± forming a rosette, subsessile, spatulate to narrowly oblanceolate, 6–21 × 0.6–2.0 cm, tapering towards the base, apex acute to obtuse, glabrous adaxially, glandular abaxially, veins invisible on both sides. Flowers solitary, axillary. Pedicel 3.0–6.0 cm long, ca. 1 mm in diameter, densely glandular. Calyx lobes lanceolate, 5–6 × ca. 3 mm, 5 (rarely 6), separate to near the base, apex acuminate, glabrous inside, glandular outside. Corolla yellow, deeply parted, tube 0.5–1.0 mm; lobes broadly ovate, 7.0 × 6.0 mm, apex obtuse, glabrous on both sides. Filaments ca. 1.5 mm long, lower 0.5 mm connate into a tube; anthers 3–3.5 mm long, ca. 1 mm in diameter, basifixed, opening by apical pores. Ovary globose, ca. 1 mm in diameter; style 2.8 mm long, slightly shorter than stamens. Capsule globose, 3.5–4 mm in diameter.

##### Phenology.

Flowering from May to June.

##### Etymology.

The new species is named after Mr. Li-Yong Fan, who first discovered and collected this rare species.

##### Distribution and habitat.

**Figure 4.**
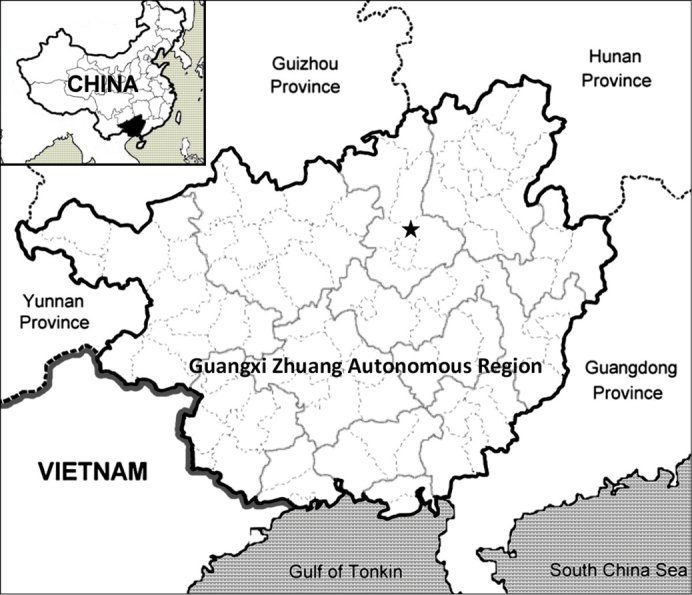
The distribution of *Lysimachia fanii* in Guangxi, China.

*Lysimachia fanii* is known only from the type locality in Taiping Town, Liucheng County, Guangxi Zhuangzu Autonomous Region, China (Fig. 4). It grows on moist limestone rock surfaces at the entrance to caves.

##### Additional specimens examined.

CHINA. **Guangxi**: Liucheng County, Taiping Town. 320 m a.s.l., 21 May 2018, *L.Y. Fan et al. FLY2018002* (IBK, GXMI).

##### Taxonomic notes.

Based on the molecular phylogeny, *L. fanii* belongs to subgenus Idiophyton, that is also supported by the morphological characters of basifixed anthers, short filaments and anthers open by apical pores. *L. fanii* is morphologically similar to *L. verbascifolia* C.M.Hu & L.K.Phan that is endemic to limestone areas in Vietnam (Phan and Hu 2011), but can be easily distinguished by its spatulate to narrowly oblanceolate leaf blade and glabrous adaxially and glandular abaxially. *L. fanii* and *L. alpestris* Champ. ex Benth. resemble each other in having congested leaves and spatulate to narrowly oblanceolate leaf blades and invisible veins and solitary inflorescences but *L. fanii* differs from *L. alpestris* by its rhizome which is branched at the apex without stolons from the base, leaf blade glabrous adaxially and glandular abaxially, basifixed anthers which open by apical pores. *L. fanii* is also similar to *L. rupestris* F.H.Chen & C.M.Hu from limestone areas distributed in south-western China and northern Vietnam (Tong et al. 2017), but it can be distinguished from the latter by its rhizome which is branched at the apex and without stolons from the base, leaf blade spatulate to narrowly oblanceolate and glabrous adaxially, lateral veins invisible on both sides. A comparison of the main characters of the four species is shown in Table 2.

**Table 2.** Comparison of characters amongst *Lysimachia fanii*, *L. verbascifolia*, *L. rupestris* and *L. alpestris*.

**Morphological traits**	***L. fanii***	***L. verbascifolia***	***L. rupestris***	***L. alpestris***
Rhizome	6–8 cm long, branched at the apex	4–10 cm long, geniculate at the base	2–5 cm long, with stolons from the base	1–4 cm long, with stolons from the base
Leaf blade	spatulate to narrowly oblanceolate, 6–21 × 0.6–2.0 cm	elliptic to broadly elliptic, 7–17 × 3.5–8.0 cm	elliptic-oblance-olate, 3–6.5 × 1.2–2.2 cm	spatulate to narrowly oblanceolate, 3–6 × 0.6–1.5 cm
Leaf indumentum	glabrous adaxially, glandular abaxially	greyish villous on both sides	minutely glandular on both sides	dense long coarse greyish hairs on both sides
Lateral veins	invisible on both sides	obvious, densely greyish villous	prominent abaxially	invisible on both sides
Inflorescence	flowers solitary	subumbellate	flowers solitary	flowers solitary
Corolla	yellow, deeply parted, tube 0.5–1.0 mm	pale yellow, divided nearly to the base	yellow, divided nearly to the base	yellow, deeply parted, tube 1–1.5 mm
Filaments	ca. 1.5 mm long, lower 0.5 mm connate into a tube	ca. 3 mm long, connate basally into a ring	ca. 1 mm long, connate basally into a ring	ca. 3 mm long, lower 1.5 mm connate into a tube
Anthers	3-3.5 mm long, basifixed, opening by apical pores	ca. 5 mm long, basifixed, opening by apical pores	4–5 mm long, basifixed, opening by apical pores	ca. 2 mm long, dorsifixed, opening by lateral slits
Flower	May to June	June to October	April to May	April

## Supplementary Material

XML Treatment for Lysimachia fanii
